# PTBP1 drives c-Myc-dependent gastric cancer progression and stemness

**DOI:** 10.1038/s41416-022-02118-5

**Published:** 2023-01-12

**Authors:** Tengyang Ni, Zewen Chu, Li Tao, Yang Zhao, Miao Zhu, Yuanyuan Luo, Masataka Sunagawa, Haibo Wang, Yanqing Liu

**Affiliations:** 1 Institute of Translational Medicine, Medical College, Yangzhou University, 225001 Yangzhou, PR China; 2 The Key Laboratory of Syndrome Differentiation and Treatment of Gastric Cancer of the State Administration of Traditional Chinese Medicine, 225001 Yangzhou, PR China; 3 Department of Pharmacy, College of Medicine, Yangzhou University, 225001 Yangzhou, Jiangsu China; 4 Department of Physiology, School of Medicine, Showa University, Tokyo, 142 Japan

**Keywords:** Cancer, Cell biology

## Abstract

**Background:**

Gastric cancer (GC) tumorigenesis and treatment failure are caused by cancer stem cells. Polypyrimidine tract binding protein 1 (PTBP1) was shown to be involved in the development of embryonic stem cells and is now being considered as a therapeutic target for tumour progression and stem-cell characteristics.

**Methods:**

PTBP1 expression in GC samples was detected using tissue microarrays. Proliferation, colony formation, spheroid formation and stem-cell analysis were used to examine PTBP1’s role in tumorigenesis and stem-cell maintenance. In AGS and HGC-27 cells with or without PTBP1 deficiency, ubiquitin-related protein expression and co-precipitation assays were performed.

**Results:**

We identified that PTBP1 was aberrantly highly expressed and represented a novel prognostic factor in GC patients. PTBP1 maintained the tumorigenic activity and stem-cell characteristics of GC in vitro and in vivo. PTBP1 directly interacts with c-Myc and stabilises its protein levels by preventing its proteasomal degradation. This is mediated by upregulating the ubiquitin-specific proteases USP28 and limiting FBW7-mediated ubiquitination of c-Myc. Moreover, the depletion of PTBP1-caused tumour regression was significantly compromised by exogenous c-Myc expression.

**Conclusions:**

By preserving the stability of c-Myc through the ubiquitin–proteasome pathway, the oncogene PTBP1 supports stem-cell-like phenotypes of GC and is involved in GC progression.

## Introduction

Despite a significant decline in incidence over the past decades, gastric cancer (GC) still accounts for most cancer-related fatalities worldwide [1, 2]. It is estimated that patients with advanced GC have a 5-year survival rate of less than 10% [3]. Therefore, understanding the molecular basis of GC is critical for improving the prognosis of GC patients. Cancer stem cells (CSCs) are found as small subpopulations of cancer cells. Due to their self-renewal and asymmetric division ability, CSCs can proliferate and form into multiple cell types in tumours, thereby leading to the continuous expansion of the tumour population [4, 5]. Importantly, CSCs are enriched in GC and gastric cancer stem cells (GCSCs) are responsible for chemoresistance and relapse after treatment [6, 7]. Therefore, there is an urgent need to fully understand the fundamental molecular pathways underlying the stemness features involved in the tumorigenic potential of GC.

Recently, the RNA-binding protein and alternative pre-mRNA splicing factor, polypyrimidine tract binding protein 1 or PTBP1, has been shown to play a critical role in cancer progression (e.g., breast, colon and bladder cancer) [8–10]. As its name suggests, PTBP1 originally binds to polypyrimidine tract regions of introns for messenger RNA processing. PTBP1 functions in all stages of mRNA metabolism, from alternative splicing, polyadenylation, and local trafficking to maturation and translation in the nucleus [11–13]. Thus, PTBP1 shapes the pattern and complexity of gene expression (e.g., pyruvate kinase) in development biology, particularly in embryonic stem cells [14]. Nevertheless, the role of PTBP1 in GC and GCSCs has not been well established to date.

Myc is a family of oncogenic transcription factors that have been well-recognised in cancer as well as CSCs [15]. As the first identified Myc family member, c-Myc coordinates different biological processes in stem cells, including proliferation, self-renewal, differentiation and cell metabolism. The expression of c-Myc is positively correlated with pluripotency markers such as Oct4, Sox2, and Nanog [16]. Interestingly, c-Myc is required for the transcriptional levels of PTBP1 [17, 18]. Thus, the regulatory relationship between c-Myc and PTBP1 is worth further investigation. Notably, c-Myc is quite unstable but frequently found to be stabilised and accumulated in cancer by both transcriptional and post-transcriptional mechanisms [16]. Whether PTBP1 can conversely fine-tune the stability of c-Myc is unknown.

In this study, we demonstrated that PTBP1 was positively correlated to the poor outcome of GC patients. Using parallel studies in two GC cell lines, we observed that the knockdown of PTBP1 attenuated the proliferation, stemness properties and xenograft growth in vivo. In contrast, overexpression of PTBP1 obtained a higher level of aggressiveness and stemness. Furthermore, PTBP1 is endogenously bound to c-Myc and primarily targeted for post-transcriptional regulation of c-Myc. Expression PTBP1 was shown to stabilise the protein levels of c-Myc while depletion of PTBP1 promoted proteasomal degradation of c-Myc via suppressing the deubiquitinating protease USP28 and upregulating the E3 ubiquitin ligase FBW7. Finally, we rescued c-Myc expression in PTBP1-deficient cells, which partially restored the cell growth and tumorsphere formation compared to the vector counterpart. Taken together, the PTBP1/c-Myc signalling axis may be an attractive target for developing new therapeutics for the treatment of gastric cancer.

## Materials and methods

### Cell lines and culture

The MKN-28, MKN-45, AGS, and HGC-27 human gastric adenocarcinoma cell lines, and the GES-1 normal human stomach epithelial cell line were obtained from the Chinese Academy of Sciences Cell Bank (Shanghai, China). Culture and PCR were used to ensure that the cell lines were clear of mycoplasma contamination, and validate the species’ provenance. The identities of cell lines were verified using STR profiling (FBI, CODIS). Cells were grown in RPMI medium supplemented with 10% foetal bovine serum (FBS) and 1% penicillin/streptomycin (Hyclone, USA). Cells were incubated at 37 °C in a humidified atmosphere containing 5% CO_2_.

### Spheroid-formation assay

In ultra-low-attachment microplates (500 cells/well in 96-well plates or 6-well plates; Corning life, cat. nos. 3474 and 3471), AGS and HGC-27 cells were grown in DMEM/F12 serum-free medium (Gibco, cat. no.: 11320033) with B27 (1:50, Gibco, cat. no.: 17504044) and N2 supplement (1:100; Gibco, cat. no.: 17502001). After 14 days of growth, cells were examined by optical microscopy (Olympus, Tokyo, Japan).

### RNAi and plasmids

The PTBP1 was knocked down in AGS and HGC-27 cells using siRNA oligonucleotides (RIBOBIO Biotechnology Co., Ltd, Guangzhou, China). The RiboFECT^TM^ CP Reagent was used to transfect cells seeded in six-well plates with 50 nM of oligonucleotides targeting PTBP1, including si-PTBP1#1 (5’-CCCUCAUUGACCUGCACAATT-3’), si-PTBP1#2 (5’-GCACAGUGUUGAAGAUCAUTT-3’), or the control vector for 48 h.

Cells were transfected with GV141-PTBP1 (Shanghai Genechem Co., Ltd, China) and Lipofectamine 2000 (Thermo Scientific, USA) for 8h and then replaced with fresh medium and incubated until 48 h to overexpress PTBP1. The efficiency of siRNA and overexpression plasmid transfection of PTBP1 was examined by qRT-PCR.

### Lentivirus transduction

To achieve long-term PTBP1 knockdown, AGS and HGC-27 cells were transduced with lentiviral-based small hairpin RNA (shRNA) targeting PTBP1 (sh-PTBP1#1: CAACGTCAAGTACAACAAT; sh-PTBP1#2: AGCCCATCTACATCCAGTT) purchased from GeneChem (Shanghai, China). Briefly, cells were grown in 24-well plates (2 × 10^4^ cells per well) and co-transduced with lentivirus and 10 μg/mL polybrene at an ideal multiplicity of infection (MOI) of 100. After puromycin (2 μg/mL) treatment for 24 h, cells were chosen and seeded for single-cell cloning. To obtain stable PTBP1- overexpressing cell lines, AGS and HGC-27 cells were transduced with the lentiviral PTBP1 or lentiviral vector using HitransG P Transfection Enhancer Reagent (Shanghai Genechem Co., Ltd, China) following the manufacturer’s instructions. Similarly, to generate c-Myc-overexpressing cell lines, AGS and HGC-27 cell lines were transduced with either lentiviral c-Myc or lentiviral vector as control.

### Tissue microarray (TMA) of GC

The GC tissue microarrays with 76 instances of GC and 82 cases of normal gastric tissues (cat. no.: HStm-Ade167Sur-01) were provided by Shanghai Outdo Biotech CO., LTD. The characteristics of patients and tumours were collected from medical records and pathology reports. Informed permission was received from the Yangzhou University, Taizhou Hospital of Zhejiang Province, and Shanghai Outdo Biotech Company. The protocols used in this study were approved by the ethical committee of the Shanghai Outdo Biotech Company, all patients gave their informed consent. Immunohistochemistry (IHC) experiments were conducted as follows. The UltraSensitive^TM^ SP (Rabbit) IHC Kit was used to stain tumour sections (4 μm) with PTBP1 rabbit monoclonal antibody (1:100, CST, cat. no.: 57246S) (MaiXin Biotechnology Company, Fuzhou, China). Differences across groups were compared using the Mann-Whitney or Kruskal–Wallis tests (SPSS Inc., Chicago, IL, USA).

### Immunohistochemistry (IHC)

First, 5-mm sections of tumour tissues were embedded in paraformaldehyde. Then, to recover antigens from deparaffinized sections, they were microwaved in 10 mmol/L citrate buffer (pH 6.0) for 15 min. After overnight incubation with PTBP1 (CST, cat. no.: 57246S), they were blocked with 5% goat serum. Next, slides were drizzled with Hastelloy’s hematoxylin (SIGMA, cat. no.: 517-28-2) for 1 min, submerged in 0.25% hydrochloric acid alcohol for 2 s, washed with tap water for 2 min and dried at room temperature. Finally, samples were analysed using a microscope digital camera system (Olympus, Tokyo, Japan).

### Cell counting kit-8 (CCK-8) assay

The viability of AGS and HGC-27 cells with PTBP1 gain or loss of function was assessed using the CCK-8 reagent (Dojindo Laboratories, Japan). First, 5 × 10^3^ cells/well were seeded onto 96-well plates. Then, they were incubated for 3 h with 10 μL of CCK-8 in a 100 μL medium per well, then analysed with an absorbance reader (PerkinElmer, USA) at 450 nm.

### Colony-formation assay

The colony formation of AGS and HGC-27 cells with PTBP1 gain or loss of function was also assessed. Briefly, 500 cells per 60-mm dish were plated for 2 weeks. To assess cell proliferation, plates were washed with PBS, fixed in 4% paraformaldehyde for 20 min, stained with 0.1% crystal violet for 30 min, and finally counted. The photos were taken in an inverted microscope (Nikon, Chiyoda-Ku, Tokyo, Japan).

### Flow cytometry

The stem-cell marker ALDH1 was identified in GC using an ALDEFLUOR kit (STEMCELL Technologies China Co., Ltd.) according to the manufacturer’s instructions. Briefly, each “test” sample tube was filled with 1.0-mL cell suspension, and the “control” tube with 5 μL ALDEFLUOR^TM^ DEAB Reagent. Then, 5 μL of activated ALDEFLUOR^TM^ Reagent was added to the “test” tube. Next, 0.5 mL of the mixture was transferred to the DEAB “control”. The “test” and “control” samples were incubated at 37 °C for 30 min. After centrifuging for 5 min at 250×*g*, the supernatant of all tubes was removed. Cell pellets were resuspended in 0.5 mL ALDEFLUOR^TM^ Assay Buffer and stored on ice or at 2–8 °C. Finally, flow cytometry was used to assess the ALDH1^+^ cells (BD LSRFortessa, USA). For side population analysis, cells were stained with 5 μg/mL Hoechst 33342 for 90 min at 37 °C. As a negative control, 50 μM verapamil (MilliporeSigma, USA) was used under the same conditions. Dead cells were eliminated with propidium iodide (Beyotime Biotechnology, China) at a final concentration of 2 μg/mL before FACS analysis. The proportion of side population cells was analysed by flow cytometry (BD LSRFortessa, USA).

### Cell proliferation EdU assay

To analyse DNA replication, cells were tagged with 5-ethynyl-2′-deoxyuridine (EdU) and evaluated using the BeyoClick^TM^ EdU-647 Kit (Beyotime Biotechnology, China) according to the manufacturer’s instructions. Cells were incubated in 10 μM EdU buffer for 2 h. Then, cells were fixed with 4% paraformaldehyde, permeabilized with 0.5% Triton X-100, then stained with the Click reaction cocktail. Next, DNA was stained with Hoechst 33342 (1 μg /mL in PBS). Finally, flow cytometry was used to identify EdU (BD LSRFortessa, USA).

### Isolation of RNA and quantitative RT-PCR

Total RNA was isolated from cells using TRIzol (Life Technology, USA). The cDNA was synthesised using the Transcriptor first-strand cDNA Synthesis Kit (Roche Technology, Inc., Swiss), according to the manufacturer’s instructions. Quantitative qRT-PCR was performed using a standard SYBR qPCR Master Mix (Roche Technology, Inc, Swiss). Results were converted to relative fold changes by the comparative threshold cycle (CT) method (the 2^-ΔΔCT^ method). Each sample was analysed in triplicate with β-actin as the internal control. Primer sequences for quantitative qRT-PCR are listed in (Supplementary Table S1).

### Western blotting (WB)

Proteins were extracted from cells using an ice-cold RIPA lysis solution supplemented with protease and phosphatase inhibitors (Thermo Fisher, USA). The protein concentration was determined using a BCA Protein Assay Kit (Beyotime Biotechnology, China) and boiled for 10 min at 100 °C. Protein lysates were separated on 10% SDS-PAGE gels, transferred to PVDF membranes (Merck Millipore, USA), and blocked for 1.5 h at room temperature with 5% skim milk. After probing with primary antibodies overnight at 4 °C, membranes were incubated for 2 h at room temperature with HRP-conjugated anti-rabbit or anti-mouse. The Image Lab software was used to enhance the chemiluminescence (ECL, Thermo Scientific, USA) and view protein bands (Bio-Rad, USA). The antibodies used were: PTBP1 (CST, cat. no.: 57246S), c-Myc (Abcam, cat. no.: ab32072), pho-c-Myc^T58^ (Abcam, cat. no.: ab185655), pho-c-Myc^S62^ (Abcam, cat. no.: ab185656), Nanog (CST, cat. no.: 4903S), Oct4 (CST, cat. no.: 2750S), Sox2 (CST, cat. no.: 3579S), FBW7 (Abcam, cat. no.: ab109617), Lamin A/C (Abcam, cat. no.: ab108595), USP28 (Abcam, cat. no.: ab126604), p21 (Abcam, cat. no.: ab109520), p27^kip1^ (Abcam, cat. no.: ab32034), Cyclin D1 (Abcam, cat. no.: ab16663) and β-actin (CST, cat. no.: 4970S).

### Nude mice xenograft model

To determine the role of PTBP1 in tumour development in vivo, we used xenograft models of HGC-27 cells with gain and loss of function of PTBP1. The Animal Care and Use Committee at Yangzhou University authorised the animal study and associated animal use procedures (AUPs). Animal studies are documented following the ARRIVE criteria for reporting animal research. Female BALB/c nude mice (4–6 weeks, SPF grade, average weight of 18 g) were provided by the Yangzhou University’s Comparative Medicine Center (Yangzhou, Jiangsu, China). Mice were housed in standardised settings (20 ± 3 °C, 40 ± 5% relative humidity), and received pathogen-free food and water. The mice were randomly divided into five groups of six rats each. Stable PTBP1 knockdown or overexpression cells were collected, resuspended in 200 μL PBS/Matrigel at an 8:1 ratio, and subcutaneously injected into the right flank of mice. After 7 days, the tumour size was determined every other day by measuring its length and width. The volume of the tumour was determined using the following formula: V = length × width^2^ × 0.5. In addition, the tumour’s size was determined by fluorescence signals via the IVIS^TM^ live imaging system (IVIS Lumina III, PerkinElmer, USA) at an excitation/emission wavelength of 580/620 nm.

### Ubiquitination assays

For in vitro ubiquitination assays, the HA-Ubiquitin plasmid was transferred into either PTBP1 knockdown or overexpressed AGS and HGC-27 cells. After 24 h, cells were incubated with 100 nM MG132 (Selleck, cat. no.: S2619) for 12 h. Then, the co-immunoprecipitation (Co-IP) assay was performed using the Pierce^TM^ Classic Magnetic IP/Co-IP Kit (Thermo Scientific, cat. no.: 88804). Briefly, cell lysates were combined with the c-Myc antibody (Thermo Scientific, cat. no.: PA5-85185) and treated overnight at 4 °C. On the next day, antigen/antibody complexes were bound to Protein A/G magnetic beads for 1 h at room temperature. Magnetic beads were washed twice with the immunoprecipitation lysis/wash buffer and once with water. After elution of antigen/antibody complexes, the WB with anti-Ubiquitin (Invitrogen, cat. no.: 701339) was performed. PTBP1 and c-Myc protein levels were also determined in whole-cell lysates (WCL).

### Immunofluorescence (IF)

First, cells were plated in six-well plates (1 × 10^4^ cells per well) on the confocal slides and incubated overnight. After washing with PBS, cells were fixed for 20 min with 4% paraformaldehyde, permeabilized for 15 min with 1% Triton X, blocked for 1 h with 1% bovine serum albumin (BSA) and incubated overnight at 4 °C with the c-Myc primary antibody (Abcam, cat.no.: ab32072), followed by Alexa-647-conjugated goat anti-rabbit secondary antibody. Then, cells were rinsed with PBS and incubated overnight at 4 °C with the primary antibody PTBP1 (Invitrogen, cat. no.: 32-4800), followed by Alexa-488-conjugated goat anti-mouse. Hoechst 33342 (1 μg/mL in PBS) was used to stain the nuclei for 15 min at room temperature. Confocal laser scanning microscopy was used to obtain the images (Carl Zeiss, LSM 880NLO).

### Statistical analyses

Results are presented as means ± standard deviations (SDs) of three independent experiments. Quantitative data were compared using two-tailed tests: Student’s *t* test or one-way analysis of variance (ANOVA) with Tukey’s or Dunnett’s post hoc tests. IBM SPSS 21.0 and GraphPad Prism 8.0 were used to conduct statistical analyses. The relation between overall survival (OS) and PTBP1 expression was assessed using the Kaplan–Meier survival curve and the log-rank test. To find independent predictive indicators, a multivariate Cox proportional hazards model was used. A two-tailed *P < 0.05 was considered statistically significant.

## Results

### PTBP1 expression is associated with poor outcomes in gastric cancer patients and is abundant in gastric cancer stem-like cells

**Fig. 1:**
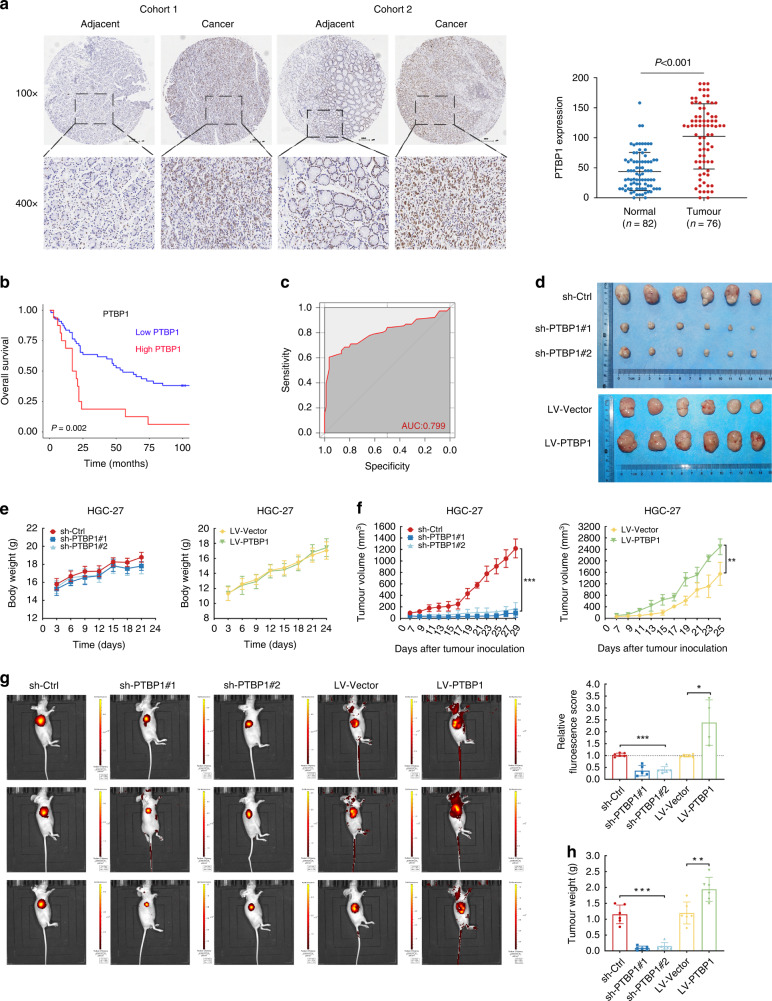
PTBP1 expression is associated with poor prognosis in gastric cancer patients and is enriched in gastric cancer stem-like cells. **a** Representative immunohistochemistry (IHC) images of PTBP1 staining on tissue microarray. PTBP1 expression is detected in gastric cancer tissues (*n* = 76) and compared with adjacent tissues (*n* = 82). *P* < 0.001. The significance of the difference was assessed by a two-tailed, unpaired *t* test. *P* value is indicated. Scale bar, 100 μm (10×). **b** Kaplan–Meier curves for Overall Survival (OS) of gastric cancer patients with high (red line) vs. low (blue line) expression of PTBP1. *P* = 0.002. **c** The receiver operating characteristic (ROC) curve for the classification of tissue samples from gastric cancer patients based on the high or low expression of PTBP1 was described with the area under the curve (AUC) = 0.799. **d** Whole xenografts in nude mice of PTBP1 knockdown or overexpressing group were imaged (*n* = 6). **e** Body weights of tumour-bearing mice were recorded every two days to exclude any adverse effects of tumour growth on the survival status of the nude mice. **f** Tumour volume in nude mice subcutaneously injected with PTBP1 knockdown or overexpressing HGC-27 cells was measured and recorded every other day starting on day 7 after inoculation of the transplanted tumours and continued for a total of 4 weeks. (*n* = 6, for each experimental group). Data represent the mean ± SD. **P < 0.01, ***P < 0.001 vs. control. Upon termination of the experiment, mice were sacrificed. **g** Representative fluorescence images of xenografts in the PTBP1 knockdown or overexpressing group were shown using IVIS™ live imaging system. Region of interest (ROI) analysis was used to quantify fluorescence signals from in vivo. The data are presented as the mean ± SD (*n* = 6). One-way ANOVA was performed. *P < 0.05, ***P < 0.001. **h** The weight of xenografts was recorded for each group. The data are presented as the mean ± SD (*n* = 6). **P < 0.01, ***P < 0.001.

To evaluate the expression profile of PTBP1 in GC patients, we performed immunohistochemical staining of PTBP1 on GC TMA containing 76 patient specimens, and 82 paired adjacent non-tumour tissues. Patient characteristics were described in Supplementary Table S2. Levels of PTBP1 expression were categorised as low (0–74%) and high (75–100%) staining intensity (Supplementary Table S3). As a result, the staining pattern of PTBP1 showed higher expression of PTBP1 in GC tissues than that in neighbouring normal tissues (Fig. 1a). Kaplan–Meier analysis and the receiver operating characteristic (ROC) curve showed higher expression of PTBP1 was significantly associated with poor overall survival (*P* = 0.002, Fig. 1b, c). Furthermore, a multivariate Cox proportional hazards model revealed that PTBP1 expression was an independent predictive factor for the poor survival of GC patients (Table 1). We also obtained the GC patients’ data from the TCGA database, and our results were consistent with public data, including PTBP1 expression levels and survival statistics (Supplementary Fig. S1A–D).

**Table 1:** Univariate and multivariate analyses of the factors correlated with the overall survival of cancer patients.

Variables	Univariate analysis				Multivariate analysis			
	*P* value	HR	95%CI		*P* value	HR	95% CI	
			Lower limits	Upper limits			Lower limits	Upper limits
Expression	**0.003**	2.554	1.369	4.762	**0.017**	2.539	1.182	5.456
Age	0.151	1.579	0.847	2.943				
Sex	0.729	1.108	0.620	1.981				
Grade	**0.045**	2.167	1.016	4.624	0.135	1.936	0.814	4.603
T stage	**0.020**	3.429	1.219	9.644	0.201	2.387	0.628	9.068
N stage	**0.009**	2.230	1.217	4.085	0.288	2.086	0.537	8.105
M stage	**0.018**	2.698	1.188	6.129	0.151	2.101	0.762	5.793
TNM stage	**0.008**	2.472	1.260	4.851	0.602	0.686	0.166	2.828
Tumour size	0.132	1.545	0.877	2.720				
Pathology Type	**0.003**	1.899	1.244	2.899	0.434	1.238	0.726	2.112

Univariate and multivariate analyses. The effect of each factor on survival was analysed using the Cox proportional risk model, and variables that were statistically significant in the univariate analysis were included in the cox multifactor survival regression analysis. Significant *P* values are shown in bold.

To investigate whether PTBP1 was involved in the stemness of GC cells, we next evaluate the correlation of PTBP1 expression with key regulatory genes of stem cells. Initially, we found that PTBP1 was universally upregulated in GC cell lines (MKN-28, MKN-45, AGS and HGC-27), compared with the normal human gastric epithelial GES-1 cell line (Supplementary Fig. S1E). Isolation of spheroid-forming cells is important to investigate cancer stem-cell-related characteristics [19], and AGS and HGC-27 cells were cultured on 3D ultra-low attachment plates as spheroids for 14 days. Surprisingly, the mRNA levels of PTBP1, together with three pluripotency factors Oct4, Nanog and Sox2 were robustly elevated in spheroid cultures than in monolayer cultures of two GC cells, which were responsible for converting cancer cells from differentiated to a stem-cell-like state (Supplementary Fig. S1F). Accordingly, we further confirmed that the protein levels of PTBP1, c-Myc, Oct4, Nanog and Sox2 were highly expressed in spheroid cultures (Supplementary Fig. S1G). Taken together, these data demonstrate that PTBP1 expression is responsible for poor prognosis in GC patients and enriched in cancer stem-like GC cells.

### PTBP1 promotes gastric cancer cell proliferation

**Fig. 2:**
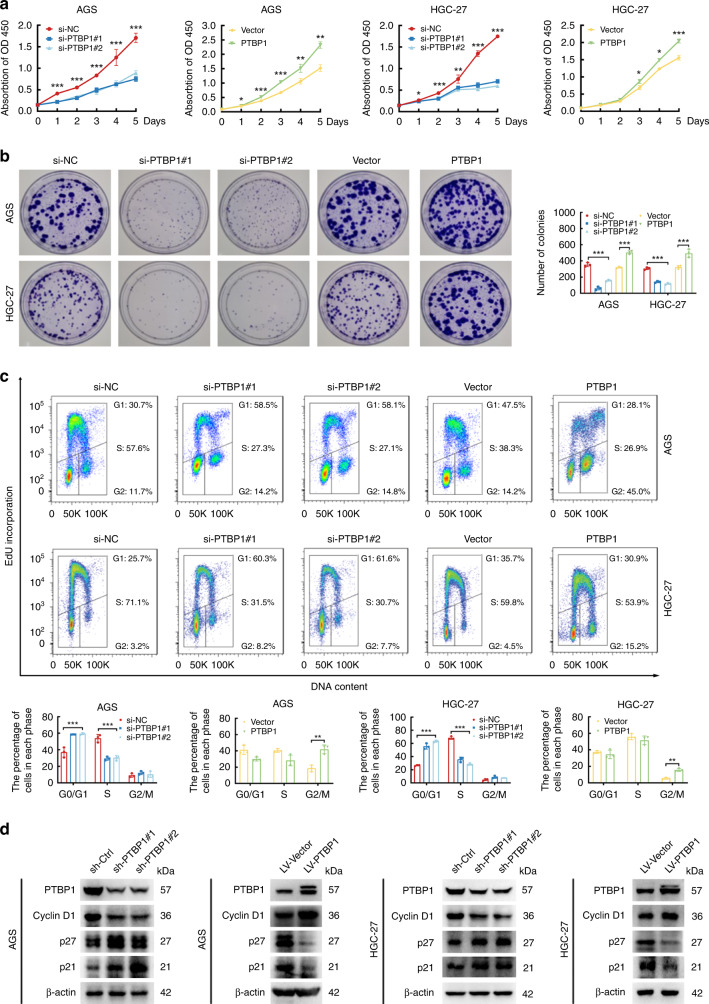
PTBP1 enhances the proliferation of gastric cancer cells. **a** PTBP1 was transient knocked down or overexpressed in AGS and HGC-27 cells and cell growth rate was detected by CCK-8 assays. The assay was carried out using dual wavelengths to detect the absorbance values of the CCK-8 reagent at a wavelength of 450 nm, at 24-h intervals for a total of 5 days. Data are representative of *n* = 3 independent experiments. **b** Colony-formation assays detect cell proliferation using crystalline violet. Images are representative of *n* = 3 independent experiments. The diameter of the dish is 60 mm. Data represent the mean ± SD ***P < 0.001 vs. control. **c** EdU assays were performed in PTBP1 knockdown or overexpressing of AGS and HGC-27 cells by flow cytometry and changes in DNA replication and cell cycle progression are observed and analysed. The rectangular box shows the cell cycle with the G1 phase at the bottom left, the G2 phase at the bottom right, and the S phase at the top. Data represent the mean ± SD of three independent experiments. **P < 0.01, ***P < 0.001 vs. control. **d** Expression of cell cycle-related proteins (Cyclin D1, p21 and p27) were detected in PTBP1 knockdown or overexpressing of AGS and HGC-27 cells by western blotting. β-actin was used as a loading control. Data are representative of *n* = 3 independent experiments. Statistical significance was determined by an unpaired two-tailed *t* test.

First and foremost, to elucidate the effect of PTBP1 on the proliferation of xenograft tumours in vivo, we conducted xenograft where 2 × 10^6^ HGC-27 cells with PTBP1 knockdown or overexpression or respective control were subcutaneously inoculated into the BALB/c nude mice. The efficiency of shRNA and overexpression lentiviral transduction of PTBP1 was examined by western blot (Fig. 2d). Initially, loss or gain of PTBP1 had no detrimental effects on the bodyweight of tumour-bearing mice (Fig. 1e). Inhibition of endogenous PTBP1 impeded the growth of xenografts as measured by tumour size. On the contrary, the expression of exogenous PTBP1 stimulated the growth of xenografts compared to the vector counterpart (Fig. 1f). All the xenografts were isolated and imaged as shown in (Fig. 1d). Meanwhile, we applied IVIS living-image system to track and quantify the growth of xenografts by the fluorescence intensity (Fig. 1g). In line with the previous results, targeting PTBP1 produced a 70% reduction in tumour weight (Fig. 1h).

To validate our above finding in vitro, we investigated the role of PTBP1 in the growth of GC cells. We generated transient knockdown expression clones using two different siRNAs (si-PTBP1 #1 and #2) and the negative control (si-NC), as well as overexpression clones of PTBP1 and the empty vector-transfected counterpart in AGS and HGC-27 cells (Supplementary Fig. S2). CCK-8 and colony-formation assays indicated that knockdown of PTBP1 decreased while overexpression of PTBP1 increased the cell proliferation potential as well as clonogenic cell survival in two GC cells (Fig. 2a, b). Next, we applied an EdU incorporation assay combined with DNA content staining to provide detailed evidence of PTBP1 in DNA replication and cell cycle progression. In line with our expectations, the knockdown of PTBP1 induced a two-fold reduction of cells in the S phase and delayed the cell cycle in the G1 phase, implying that inhibition of PTBP1 could suppress the DNA synthesis in GC cells due to defective G1/S transition. Interestingly, overexpression of PTBP1 in AGS cells caused the emergence of polyploid cells within the S phase, accompanied by an accumulation of cell proportion in the G2 phase. However, we did not observe a significant accumulation of polyploids in HGC-27 cells, suggesting that PTBP1 was necessary for the heterogeneity of GC cells (Fig. 2c). Activation of cell cycle checkpoints reflects a reduction of the cyclins and induction of the inhibitors of cyclin-dependent kinases. As silencing of PTBP1 led to the blockage of G1/S transition, we additionally explored the alterations of Cyclin D1, p21 and p27 that are involved in the G1/S cell cycle control. Consistently, knockdown cells showed an impaired expression of Cyclin D1, whereas the expression of p21 and p27 were enforced. Conversely, PTBP1 overexpressed cells showed an augmented expression of Cyclin D1, whereas the expression of p21 and p27 were attenuated (Fig. 2d).

Migration is prerequisites for cancer cell metastasis. We simultaneously examined the effects of PTBP1 on the phenotypic change using High-Content Imaging System Analysis. Similarly, knockdown of PTBP1 was mitigated while overexpression of PTBP1 increased cell migration of two GC cells (Supplementary Fig. S3). Collectively, these results further support the notion that PTBP1 is a tumour promoter of GC cells both in vitro and in vivo.

### PTBP1 is critical for gastric cancer stem-like properties

**Fig. 3:**
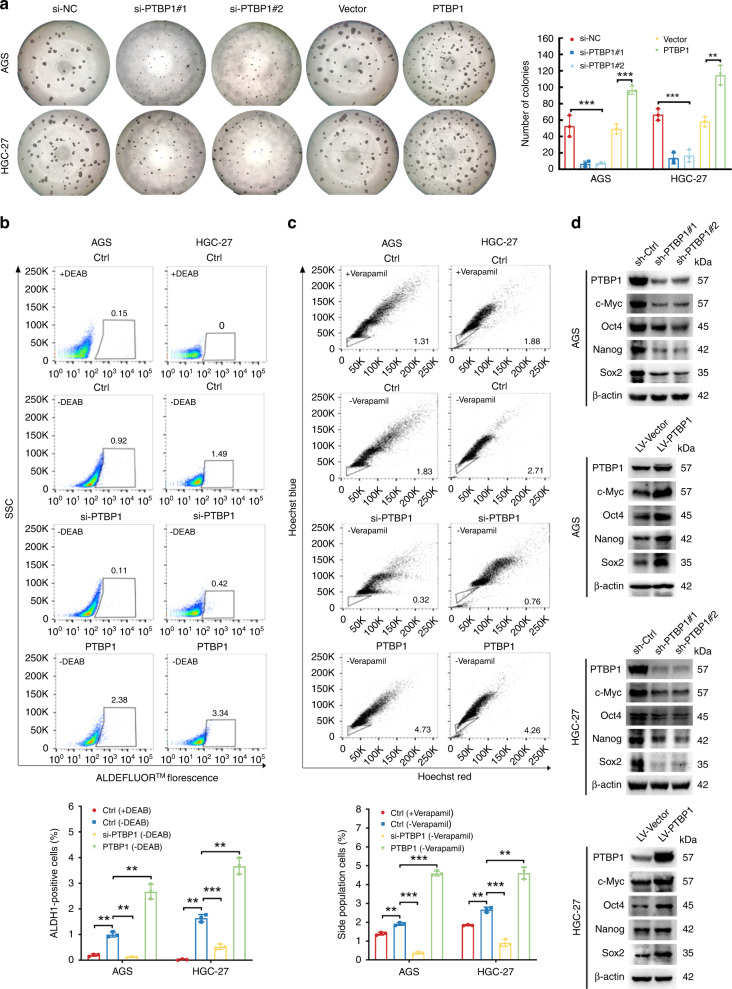
Overexpression of PTBP1 promoted cancer stem-like properties. **a** Representative images of spheroid formation in PTBP1 knockdown or overexpressing of AGS and HGC-27 cells, clusters of tumorspheres larger than 50 μm in diameter were included in the spheroid count. Each well of the 96-well plate has a diameter of 6.94 mm. Data are representative of *n* = 3 independent experiments. **P < 0.01, ***P < 0.001 vs. control. **b** ALDH1 activity in PTBP1 knockdown or overexpressing of AGS and HGC-27 cells was analysed by flow cytometry. Cells in the polygon are positive target cell populations that have released the ALDH1 enzyme. Data represent the mean ± SD of three independent experiments. **P < 0.01, ***P < 0.001 vs. control. **c** Flow cytometry analysis shows side populations in PTBP1 knockdown or overexpressing AGS and HGC-27 cells. Data represent the mean ± SD of three independent experiments. **P < 0.01, ***P < 0.001 vs. control. **d** Expression of cancer stem-like properties proteins (c-Myc, Oct4, Nanog and Sox2) was detected in PTBP1 knockdown or overexpressing of AGS and HGC-27 cells by western blotting. β-actin was used as a loading control. Data are representative of *n* = 3 independent experiments. Statistical significance was determined by an unpaired two-tailed *t* test.

Given that PTBP1 was upregulated in the stem-like properties of GC cells in the spheroid-formation assay. We next genetically validated whether PTBP1 was essential for the maintenance of stemness of GC cells. In agreement, PTBP1 knockdown markedly repressed the spheroid formation of two GC cells, and this effect could be strengthened by PTBP1 overexpression (Fig. 3a). Aldehyde dehydrogenase or ALDH functions as an epithelial marker of gastric cancer stem cells [20]. ALDH activity was measured by a flow cytometer using the ALDEFLUOR™ fluorescent reagent system. In the tested GC cell lines, the percentage of ALDH-positive cells was diminished by PTBP1 knockdown and multiplied by PTBP1 overexpression (Fig. 3b). Since a small fraction of side population (SP) cells represent a subset of cancer stem cells, we detected the presence of SP cells by flow cytometry analysis. Consistently, our data further confirmed that PTBP1 contributes to the proportion of SP cells (Fig. 3c).

Next, we detected key transcription factors associated with cancer stem-cell phenotypes after genetic manipulation of PTBP1. In addition to Oct4, Nanog, and Sox2, we additionally detected c-Myc as we introduced. Western blot assay showed that knockdown of PTBP1 significantly decreased the protein levels of Oct4, Nanog, Sox2 and c-Myc, while GC cells transfected with PTBP1 exhibited higher levels of all these transcription factors (Fig. 3d). Altogether, these data indicate that PTBP1 is an essential modulator for the maintenance of gastric cancer stem-cell properties.

### PTBP1 stabilises c-Myc at the post-transcriptional level

**Fig. 4:**
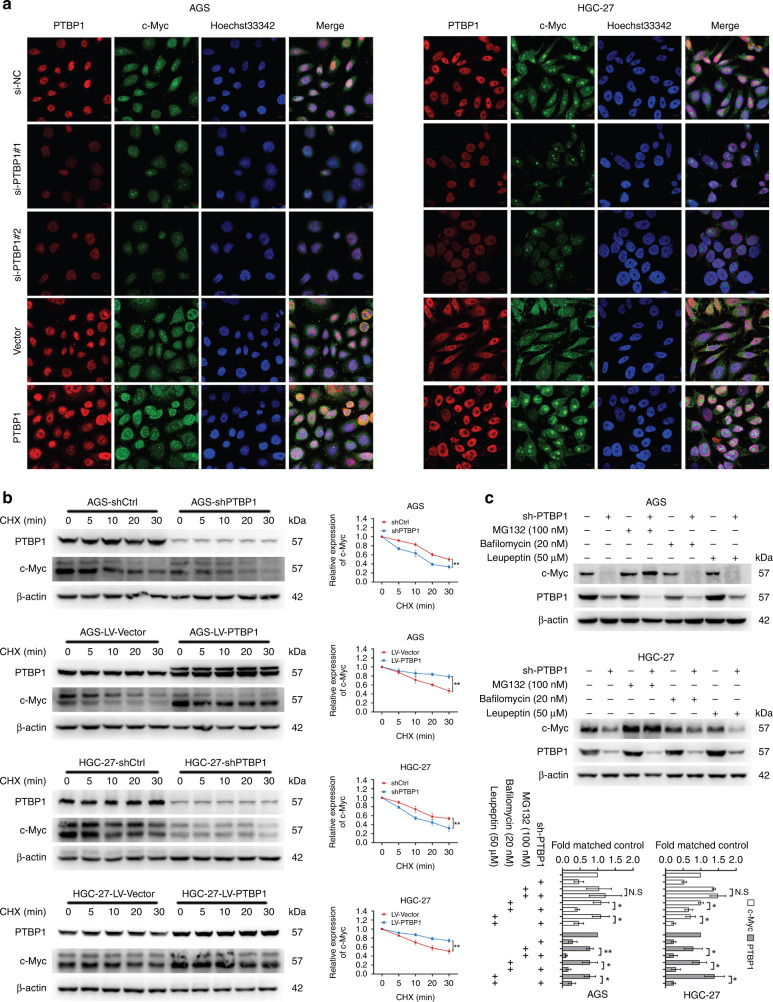
PTBP1 enhances c-Myc stability at a post-transcription level. **a** Immunofluorescence assays were conducted to detect the expression of c-Myc in AGS and HGC-27 cells with PTBP1 knockdown or overexpressing (PTBP1 in red; c-Myc in green; Hoechst 33342 in blue; scale bar: 50 μm). Data are representative of *n* = 3 independent experiments. **b** AGS and HGC-27 cells with or without PTBP1 knockdown or overexpression were treated with CHX (100 μg/ml) for the indicated times. The half-life of c-Myc was measured by western blotting. β-actin was used as a loading control. The data are presented as the mean ± SD of three independent experiments. **P < 0.01, ***P < 0.001 vs. control. Statistical significance was determined by an unpaired two-tailed *t* test. **c** Total protein expression of c-Myc in sh-Ctrl or sh-PTBP1 GC cells with the presence or absence of MG132 (100 nM), Bafilomycin (20 nM), and Leupeptin (50 μM) for 12 h was performed by western blotting. β-actin was used as a loading control. The data are presented from three independent experiments.

Since c-Myc is highly unstable [21], we further set out to understand how PTBP1 altered the expression of the c-Myc. As c-Myc is predominantly localised in the nucleus, parallel immunofluorescence staining of c-Myc further revealed that inhibition of PTBP1 scavenged while activation of PTBP1 promoted the nuclear signal of c-Myc (Fig. 4a). Given that the mRNA levels of c-Myc were not affected by PTBP1 (Supplementary Fig. S4), we then examined the effects of PTBP1 on c-Myc protein stability. In the presence of the protein synthesis inhibitor cycloheximide (CHX), we observed that knockdown of PTBP1 shortened while overexpression of PTBP1 extended the half-life of c-Myc, suggesting that PTBP1 regulated c-Myc expression via a post-translational mechanism (Fig. 4b). Next, we asked whether PTBP1 could prevent the degradation of c-Myc. We found that the proteasome inhibitor MG132, rather than late-phase autophagy inhibitor bafilomycin A1 or protease inhibitor leupeptin, successfully induced c-Myc protein turnover (Fig. 4c). Therefore, the proteasomal pathway is involved in c-Myc degradation upon PTBP1 inhibition.

### PTBP1 prevents c-Myc degradation from the ubiquitin pathway

**Fig. 5:**
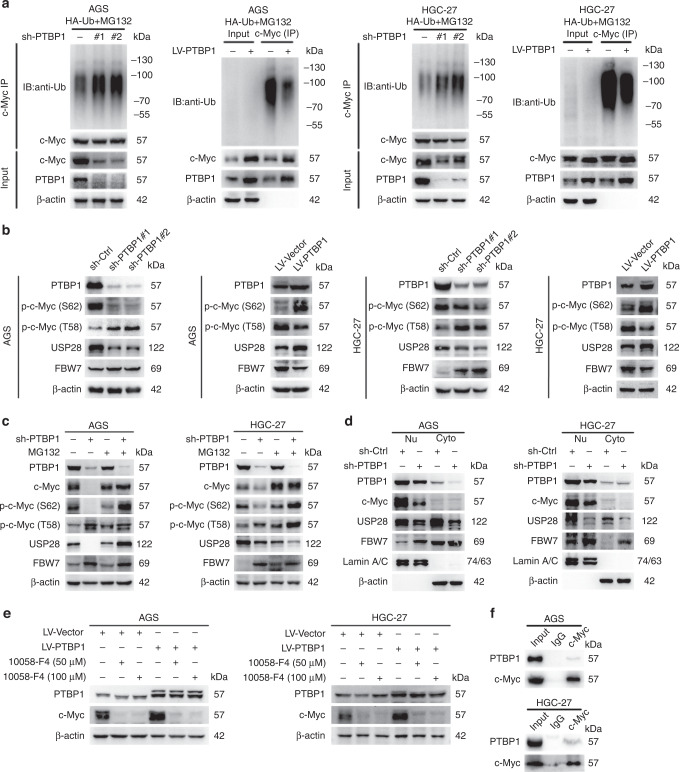
PTBP1 interacts with c-Myc and stabilises the c-Myc protein through the ubiquitin–proteasome system. **a** Ubiquitin plasmid was transfected into AGS and HGC-27 cells with or without PTBP1 knockdown or overexpression and then MG132 (100 nM), was treated for 12 h, following total protein was immunoprecipitated with c-Myc antibody, and the ubiquitination level of c-Myc was detected by western blotting. β-actin was used as a loading control. The data are presented from three independent experiments. **b** Effect of PTBP1 knockdown or overexpressing on p-c-Myc (S62), p-c-Myc (T58), USP28 and FBW7 in AGS and HGC-27 cells by western blotting. β-actin was used as a loading control. The data are presented from three independent experiments. **c** AGS and HGC-27 cells with or without PTBP1 knockdown were exposed to MG132 (100 nM) for 12 h, then c-Myc, p-c-Myc (S62), p-c-Myc (T58), USP28 and FBW7 proteins expression were detected by western blotting. β-actin was used as a loading control. The data are presented from three independent experiments. **d** Nuclear and cytoplasmic extracts from AGS and HGC-27 cells with or without PTBP1 knockdown was obtained for detecting PTBP1, c-Myc, USP28 and FBW7. Lamin A/C was used as an internal standard for the nucleus and β-actin was used as an internal control for cytoplasm. The data are presented from three independent experiments. **e** AGS and HGC-27 cells with or without PTBP1 knockdown were exposed to a specific c-Myc inhibitor 10058-F4 (50, 100 μM) for 24 h, suppression efficiency was detected by western blotting. β-actin was used as a loading control. The data are presented from three independent experiments. **f** Anti c-Myc antibodies or control IgG were used to immunoprecipitate lysates in AGS and HGC-27 cells. Anti-PTBP1 was detected by western blotting. The data are presented from three independent experiments.

Several ubiquitin ligases and deubiquitinases have been shown to control the stability of c-Myc [22]. We then hypothesised that PTBP1 protected c-Myc stability in proteasome-dependent degradation. To this end, we carried out an in vitro ubiquitination assay to test this possibility. Indeed, PTBP1 knockdown reduced c-Myc stability by increased ubiquitination. On the contrary, overexpression of PTBP1 rescued c-Myc protein from ubiquitin-mediated degradation (Fig. 5a). Generally, c-Myc phosphorylation on Thr-58 residue causes rapid proteolysis by the ubiquitin pathway, while phosphorylation on Ser-62 represents a stable form of c-Myc [23]. Evidence has shown that E3 ubiquitin ligases such as FBW7 and β-Trcp are responsible for c-Myc ubiquitination, whereas deubiquitinases such as USP28 and USP36 can antagonise the ubiquitin-dependent degradation of c-Myc [24–27]. We further observed that PTBP1 deficiency enhanced FBW7 level and c-Myc phosphorylation on Thr-58, while decreased USP28 expression and c-Myc phosphorylation on Ser-62. Moreover, overexpression of PTBP1 had the opposite effects (Fig. 5b). Importantly, the above results were partially reversed by MG132 treatment (Fig. 5c), indicating the ubiquitination and deubiquitination of c-Myc were involved in PTBP1-mediated stabilisation of c-Myc.

Besides, we identified that PTBP1 primarily prevented c-Myc proteolysis in the nuclear compartments, and PTBP1 deletion impaired USP28 expression in the nuclear fraction, implying that PTBP1 triggered a rapid protective mechanism from c-Myc degradation (Fig. 5d). Notably, we also utilised a well-known c-Myc inhibitor 10058-F4, which completely reversed PTBP1-mediated c-Myc accumulation (Fig. 5e). Next, we questioned whether PTBP1 could physically interact with c-Myc. We performed co-immunoprecipitation analysis for endogenous PTBP1- c-Myc interaction. Indeed, our data demonstrated that constitutive PTBP1 was able to bind c-Myc in two GC cells (Fig. 5f), meaning that PTBP1 might directly form a complex with c-Myc and preserve it from degradation. Altogether, these data provide strong support for the notion that PTBP1 interacts with c-Myc and regulates its stability through the ubiquitin–proteasome pathway.

### c-Myc is required for PTBP1-dependent cancer stem-like properties

**Fig. 6:**
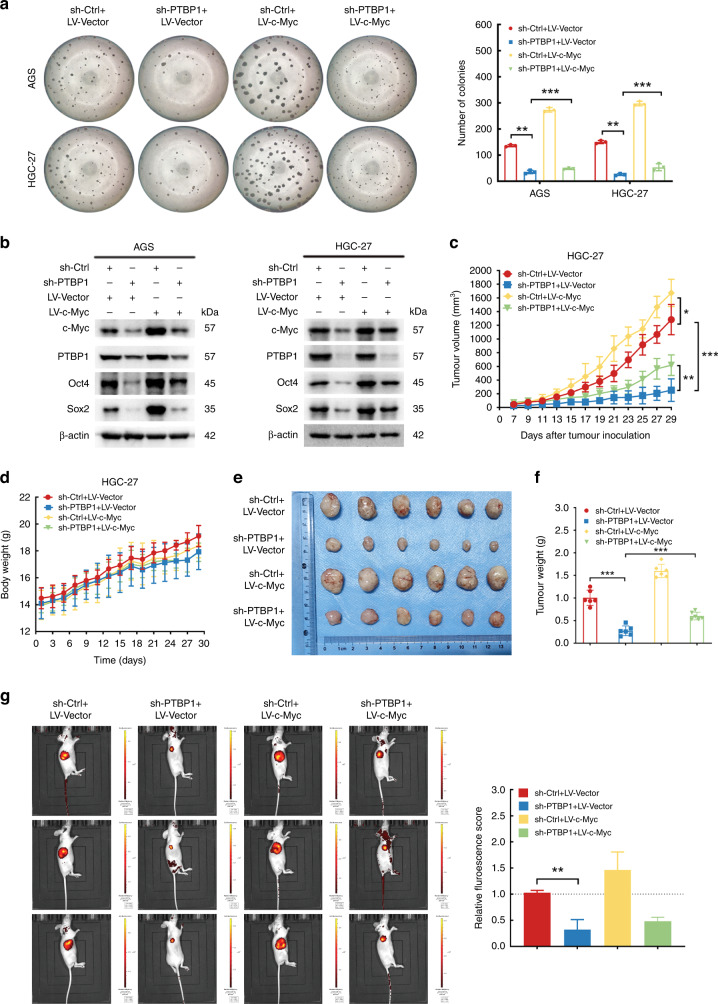
c-Myc is required for PTBP1-dependent cancer stem-like properties. PTBP1 was knocked down in AGS and HGC-27 cells with or without overexpressing c-Myc. **a** Sphere-forming abilities were detected. Images are representative of *n* = 3 independent experiments. The diameter of the well is 6.94 mm. The data are presented as the mean ± SD. **P < 0.01, ***P < 0.001. **b** The c-Myc, Oct4 and Sox2 protein expression levels were analysed by western blotting. β-actin was used as a loading control. The data are presented from three independent experiments. Xenograft tumour model in vivo was conducted using PTBP1 knockdown or vector-transfected HGC-27 cells combined with c-Myc overexpression (*n* = 6 in each group). **c** Tumour volume in nude mice subcutaneously injected with PTBP1 knockdown or vector-transfected HGC-27 cells combined with c-Myc overexpression was measured and recorded every other day starting on day 7 after inoculation of the transplanted tumours and continued for a total of 4 weeks. (*n* = 6, for each experimental group). Data represent the mean ± SD. **P < 0.01, ***P < 0.001 vs. control. Upon termination of the experiment, mice were sacrificed. **d** Body weights of tumour-bearing mice were recorded for 30 days to exclude any adverse effects of tumour growth on the survival status of the nude mice. **e**, **f** When the mice were sacrificed, whole tumour size and the weight of tumours were calculated for each group (*n* = 6). The data are presented as the mean ± SD. *P < 0.05, **P < 0.01, ***P < 0.001. **g** Representative fluorescence images were shown using IVIS™ live imaging system. Region of interest (ROI) analysis was used to quantify fluorescence signals in vivo. The data are presented as the mean ± SD. **P < 0.01.

To further demonstrate whether PTBP1 facilitates GC stem-cell phenotypes in a c-Myc-dependent manner, we further restored c-Myc expression in PTBP1 knockdown AGS and HGC-27 cells. As we anticipated, c-Myc expression compromised PTBP1 depletion-induced inhibition of tumorsphere formation, compared to PTBP1 knockdown alone (Fig. 6a). Also, PTBP1 knockdown-induced reduction of stem-cell factors was partially rescued by c-Myc expression (Fig. 6b). Moreover, the expression of c-Myc also attenuated the PTBP1 knockdown-mediated inhibitory effect of xenograft growth, as measured by tumour volume (Fig. 6c), tumour size (Fig. 6e), tumour weight (Fig. 6f), and fluorescence intensity of xenograft (Fig. 6g). However, there was no effect on bodyweight (Fig. 6d). In addition, a high-throughput cell migration assay and colony-formation assays also confirmed that c-Myc was responsible for PTBP1-induced cell migration (Supplementary Fig. S5). Overall, these findings strongly demonstrate that c-Myc is required for PTBP1 as an upstream regulator for maintaining cancer stem-like properties of GC cells.

## Discussion

Gastric cancer is the fifth most diagnosed cancer and the third leading cause of cancer-related mortality worldwide [28]. Despite recent advances in diagnostic capability and treatment techniques, the chance of recurrence and metastasis in advanced GC patients remains high, as well as their prognosis [29, 30]. Increasing evidence has shown that CSC participates in cancer initiation, recurrence, metastasis, and treatment resistance [31, 32].

The CSCs hypothesis states that a minority of cells in cancer tissue can self-regenerate, proliferate and have multiple differentiation potentials. CSCs give rise to different phenotypes of tumour cells, which contribute to tumour heterogeneity and therefore play a decisive role in tumorigenesis and progression. In addition, they become resistant during radiotherapy and chemotherapy and rapidly migrate and metastasize into new tumours [33–35].

Therefore, it is critical to understand how GCSCs retain stem-like features and to identify GCSC-related therapy options that might assist improve the prognosis of GC patients. Our findings demonstrated that PTBP1 expression was significantly increased in GCSCs and GC tissues. This increased expression was significantly associated with bad outcomes in GC patients. Hence, PTBP1 can be a prognostic factor of GC. These findings also supported the hypothesis that PTBP1 is a GC oncogene.

Through the alternative splicing of exons, PTBP1 is involved in tumour EMT [36], glycolysis [37] and metabolic reprogramming [38]. Recent studies have shown that PTBP1 plays a vital role in the maintenance of cancer stem-cell phenotypes [39] and alleviating chemotherapy resistance [40]. However, the role of PTBP1 in GC has not been fully explored previously and its potential mechanisms remain unknown. In the present study, we showed that PTBP1 can promote the proliferation of GC cells in vitro and accelerate the growth of tumours in vivo. Furthermore, the depletion of PTBP1 restricted cell proliferation, induced cycle arrest, and affected the expression of related cyclins. Interestingly, the cell cycle changes promoted by PTBP1 overexpression were not opposed to PTBP1 depletion but led to an accumulation of cells in the G2 phase. Similar studies have shown that knockdown of PTBP1 causes an increase in the proportion of S-phase cells [41], and PTBP1 enhances the internal ribosomal entry site-dependent translation of p27^Kip1^ mRNA and regulates the transition from G1 to S-phase [42]. However, it has also been reported that knockdown of PTBP1 enhances the transcriptional activity of the cell cycle-dependent kinase (CDK) inhibitors p21 and p27 [43]. We prefer the latter view because of our findings that the knockdown of PTBP1 decreases the number of S-phase cells and upregulates the expression of p21 and p27. Since S-phase is the main period of cell proliferation, a decrease in the proportion of cells in the S-phase usually reflects a reduction in cell proliferation capacity, which is consistent with our results. Support for our results also includes the increased demand for PTBP1 in the late S phase [44], indicating that the overexpression of PTBP1 can promote the S to G2 phase transition and accelerate the cell cycle. Furthermore, PTBP1 deficiency causes an increase in PTBP2 expression, as well as other regulatory layers intervening to compensate for some of the functions of PTBP1 [45]. Therefore, we believe that PTBP1 promotes cell growth by stimulating cell cycle progression. The inconsistency in study reports may be due to differences in the types of cells, the sites or method by which PTBP1 was knocked down.

Given the possible relationship between PTBP1 and GCSCs, we analysed whether PTBP1 affects cancer stem-cell-like phenotypes. As expected, we showed that the depletion of PTBP1 impaired cancer stem-cell-like phenotypes, including spheroidization, clonogenicity, the expression of CSC markers. In contrast, PTBP1 overexpression preserved cancer stem-cell-like characteristics. Besides mediating tumour cell invasion and metastasis, the EMT is closely related to cancer stem-cell-like phenotypes. Increasing studies have shown that EMT plays a vital role in enriching cells with CSC properties and chemoresistance. Hence, we showed the downregulation of migratory abilities in PTBP1-knockdown cells using a high-content imaging system, whereas PTBP1 overexpression led to the opposite results. Overall, we indicated that PTBP1 participates in the maintenance of cancer stem-cell-like properties.

Further, to reveal the underlying mechanisms of PTBP1 in maintaining CSC-like properties, we examined important transcription factors that have been reported to regulate CSC-like phenotypes and determined that c-Myc is responsible for the regulation of CSC-like phenotypes by PTBP1. Moreover, PTBP1 upregulated c-Myc expression at the protein level rather than mRNA level. This suggested that PTBP1 regulated c-Myc expression at the post-transcriptional level. This is consistent with the findings of Cobbold et al. [46]. Then, we analysed the proteasome, lysosomal, and autophagy pathways to determine how c-Myc was degraded. We found that PTBP1 maintained the stability of c-Myc through the proteasome pathway.

For the current clinical utilisation of PTBP1, Qian et al. [47] in 2020 described an antisense oligonucleotide that inhibits PTBP1 and converts astrocytes into new neurons, which identifies a potentially and powerful new clinically viable approach to treat neurodegenerative diseases such as Parkinson’s by replacing lost neurons. Another recently developed RNA-targeted CRISPR system, CasRx, for in vivo viral delivery, downregulates PTBP1 and enables efficient conversion of Müller glial cells to retinal ganglion cells (RGCs), thereby alleviating disease symptoms associated with RGC loss. Glial-to-neuronal conversion by CasRx-mediated Ptbp1 knockdown represents a promising in vivo genetic approach for the treatment of various diseases caused by neuronal deficiency [48]. In the field of oncology research, PT109, a novel multikinase inhibitor, reprogrammes glioblastoma multiforme (GBM) into oligodendrocytes by decreasing the level of PTBP1 and increasing the ratio of pyruvate kinase M1/2 (PKM1/2), and alters the metabolic pattern of GBM via the PTBP1/PKM1/2 pathway [49].

However, PTBP1 inhibitors are still in a vacant state in gastric cancer treatment, and our results confirm that PTBP1 is critical to the progression of gastric cancer, therefore PTBP1 inhibitors suitable for gastric cancer targets are urgently exploited. Classical therapeutic targets of tumours involved in PTBP1 include HER2 [50], STAT3 [51] etc., and inhibitors against these targets have been developed. In addition, PTBP1 is also involved in the development of multidrug resistance in tumours. In the clinical treatment of breast cancer, H3K27ac modification-induced upregulation of lncRNA ZNF649-AS1 gene may lead to autophagy and trastuzumab resistance by binding to PTBP1 and promoting ATG5 transcription [52]. Thus, the development of PTBP1 inhibitors has a potential synergistic effect on reducing tumour resistance to targeted drugs, yet these studies represent another major gap in the field of gastric cancer, and our study attempts to break through the limitations of PTBP1 in the therapeutic field of gastric cancer. Although no PTBP1 inhibitors suitable for gastric cancer treatment were identified, our findings demonstrate that PTBP1 exerts antitumor effects can be mediated through downstream c-Myc, which is one of the key targets for gastric cancer treatment, not only promoting gastric cancer progression and lung metastasis [53], but also involved in the induction of PD-L1 expression [54]. Therefore, the use of c-Myc inhibitors provides ideas for the treatment of gastric cancer and reduction of drug resistance until inhibitors of PTBP1 for gastric cancer are available. However, more work is still being needed to translate basic research results into practical clinical applications, but in light of the progress of PTBP1 inhibitors in the field of neurology, there are great prospects for the development of PTBP1 inhibitors in the field of tumour therapy.

Since c-Myc is an unstable protein, it is targeted for proteasomal degradation by a large number of ubiquitin ligases [55]. The two major phosphorylation sites of c-Myc are at the N-terminal of amino acids ser-62 (S62) and thr-58 (T58), which might cooperate to control the protein stability. The phosphorylation of c-Myc at S62 contributes to the stability, while phosphorylation at T58 contributes to its degradation [56]. The F-box and WD repeat domain-containing 7 (FBW7) is an E3 ubiquitin ligase that catalyzes the degradation of c-Myc by the proteasome. Before binding to FBW7, c-Myc must be dephosphorylated at S62, then phosphorylated at T58 for protein breakdown [57–59]. In addition, the ubiquitination of c-Myc is inhibited by the deubiquitinase USP28, which might be carried on FBW7 and operate as a c-Myc protein stabiliser [60]. Thus, the E3 ubiquitin ligase FBW7 and the deubiquitinases USP28 cooperate to maintain the stability of c-Myc. We showed that overexpression of PTBP1 prolonged the half-life of c-Myc after treatment with cycloheximide (CHX), but the reduction of PTBP1 accelerated its degradation. In addition, treatment with MG132 partly inhibited c-Myc phosphorylation at Ser-62 and Thr-58, indicating that PTBP1 inhibits c-Myc degradation through the ubiquitin–proteasome system, thereby increasing c-Myc stability. Finally, we demonstrated that the overexpression of c-Myc reversed the drop in CSC phenotypes induced by PTBP1 depletion in vitro and in vivo.

Uncertainly, PTBP1 may be a downstream molecule induced by c-Myc [61] or it may act as an upstream, regulating c-Myc expression by binding to the IRES region of c-Myc [46]. In this study, the c-Myc synthesis inhibitor 10058-F4 was unable to restrict PTBP1 expression, which makes us more supportive of the latter hypothesis. It is possible that the reason for the discrepancy in these findings is the existence of a parallel relationship between PTBP1 and c-Myc, and the intermediate feedback loop needs to be filled by further findings. Altogether, our findings strongly suggested that c-Myc is essential for PTBP1 to sustain GC cancer stem-cell-like characteristics.

## Supplementary information

Reproducibility Checklist

Supplementary

Figure S1

Figure S2

Figure S3

Figure S4

Figure S5

## Data Availability

The data that support the findings of this study are available upon request from the corresponding author. The data are not publicly available due to privacy and ethical restrictions.
