# Supplementary material for: PTBP1 drives c-Myc-dependent gastric cancer progression and stemness
Source: Br J Cancer. 2023 Jan 12;128(6):1005–18. doi: 10.1038/s41416-022-02118-5 (PMC10006230; doi:10.1038/s41416-022-02118-5)
Supplement: Supplementary file 2 — Supplementary [file 41416_2022_2118_MOESM2_ESM.pdf]

# Supplementary

## PTBP1 drives c-Myc-dependent gastric cancer progression and stemness

Tengyang Ni[1,2], Zewen Chu[1,2], Li Tao[1,3], Yang Zhao[1,3], Miao Zhu[1,2], Yuanyuan Luo[1,2], Masataka Sunagawa[4], Haibo Wang[1,2], and Yanqing Liu[1,2]*

*[1]Institute of Translational Medicine, Medical College, Yangzhou University, Yangzhou, 225001, PR China.*

*[2]The Key Laboratory of Syndrome Differentiation and Treatment of Gastric Cancer of the State Administration of Traditional Chinese Medicine, Yangzhou, 225001, PR China.*

*[3]Department of Pharmacy, College of Medicine, Yangzhou University, Yangzhou, Jiangsu, 225001, China.*

*[4]Department of Physiology, School of Medicine, Showa University, Tokyo 142, Japan.*

## SUPPLEMENTAL INFORMATION

-Supplemental materials and methods

-5 supplemental figures with legends

-3 supplemental tables

## Supplemental materials and methods

*High-Content Imaging System Analysis*

Cells were seeded at a density of $3\times10^3$ cells per well onto 96-well plates and cultured for 24 hours while cells were growing exponentially. Plates were then filmed for 12 hours in a PerkinElmer CLS High Content Imaging Analysis System. High-content imaging was performed using an Opera Phenix (Perkin Elmer, USA), using a 40× lens. 9 fields of view were imaged for each well, with 10 z-stacks per field at 0.1 μm intervals to ensure comprehensive imaging of the cell monolayer. Triplicate biological and technical replicates were performed for all experiments. Data collection and analysis were performed using the Harmony 4.1 software (PerkinElmer, Waltham, Massachusetts, USA). An optical correction was performed using flat-field and bright-field correction.

## Supplementary figures

**Fig S1. PTBP1 expression is associated with poor prognosis in gastric cancer patients in TCGA Cohorts**

(**A&B**) In the TCGA Cohorts, PTBP1 expression and paired PTBP1 expression were detected in stomach tumor tissues (red, n=408) and compared to normal tissues (blue, n=36). (**C**) Kaplan-Meier curves for Disease-Free Survival (DFS) of gastric cancer patients with high PTBP1 (black line, n=120) vs. low PTBP1 (red line, n=121) expression in the TCGA database. *P*=1e+00 (**D**) Kaplan-Meier curves for Overall Survival (OS) of gastric cancer patients with high PTBP1 (red

line, n=183) vs. low PTBP1 (black line, n=183) expression in the TCGA database. *P*=5.4e-01. (**E**) The expression levels of PTBP1 protein in GC cell lines and normal gastric cell line GES-1 were investigated using western blotting, and β-actin was used as a loading control. WB Data are representative of three independent experiments. (**F&G**) The expression levels of PTBP1, Oct4, Nanog, and Sox2 between monolayer and spheroid were detected by qRT-PCR assays and western blotting. β-actin was used as a control. Data from qRT-PCR are presented as mean ± SD from three independent experiments. *$p < 0.05$, **$p < 0.01$, *** $p < 0.001$ vs. control. WB data are representative of three independent experiments. Statistical significance was determined by an unpaired two-tailed *t*-test.

**Fig. S2. The efficiency of siRNA and overexpression plasmid transfection of PTBP1**

PTBP1 was knocked down using si-RNA or overexpressed by plasmid in AGS and HGC-27 cells. The mRNA expression levels of PTBP1 were analyzed by qRT-PCR. β-actin was used as a control. Data are presented as mean ± SD of three independent experiments. * $p < 0.05$, ** $p < 0.01$, *** $p < 0.001$ vs. controls.

**Fig. S3. PTBP1 promotes cell invasion and migration**

(**A&B**) High-Content Imaging System Analysis showing the migration ability of AGS and HGC-27 cells with knockdown or overexpression of PTBP1. Data are presented as mean ± SD of three independent experiments. ** $p < 0.01$, *** $p$ <

0.001 vs. controls.

**Fig. S4. The mRNA levels of c-Myc were not affected by PTBP1**

(**A&B**) Quantitative RT-PCR was used to detect c-Myc mRNA expression in PTBP1 knockdown or overexpressed AGS and HGC-27 cells. β-actin was selected as the internal control. Data are presented as mean ± SD (n = 3). Data were analyzed using a two-tailed Student's *t*-test. * $p < 0.05$, ** $p < 0.01$, *** $p < 0.001$ vs. controls.

**Fig. S5. c-Myc is required for PTBP1-facilitating cancer stem-like properties**

(**A**) PTBP1 was knocked down with or without c-Myc overexpression in AGS and HGC-27 cells, and the colony formation ability was detected. Data are presented as mean ± SD. ** $p < 0.01$, *** $p < 0.001$. (**B**) The trajectory of cell migration was tracked by High-Content Imaging System and photographed every 30 minutes for 12 hours, and the mean square of migration displacement was calculated to show the cumulative sum of cell migration distance. (**C**) Representative image of cell migration trajectory at the endpoint, and visualization of the displacement trajectory of the relative motion of individual cells.

## Supplementary tables

**Table S1. Primers sequences for qPCR.**

| Genes Names | Sequence 5'-3' |
|---|---|
| PTBP1 Forward | CCAGAACATCTTTCCACCCTCAGC |
| PTBP1 Reverse | CAGTGCCATCTTGCGGTCCTTC |
| c-Myc Forward | CGAGGAGAATGTCAAGAGGCGAAC |
| c-Myc Reverse | GCTTGGACGGACAGGATGTATGC |
| Nanog Forward | AGATGCCTCACACGGAGACTGTC |
| Nanog Reverse | TGGGTTGTTTGCCTTTGGGACTG |
| Sox2 Forward | CAGCATGTCCTACTCGCAGCAG |
| Sox2 Reverse | CTGGAGTGGGAGGAAGAGGTAACC |
| Oct4 Forward | GTGGTCCGAGTGTGGTTCTGTAAC |
| Oct4 Reverse | CCCAGCAGCCTCAAAATCCTCTC |
| β-actin Forward | CATGTACGTTGCTATCCAGGC |
| β-actin Reverse | CTCCTTAATGTCACGCACGAT |

**Table S2. Correlation between PTBP1 expression and clinicopathological characteristics.**

| | variables | PTBP1 expression | | total | $\chi^2$ | p-value |
|---|---|---|---|---|---|---|
| | | low | high | | | |
| Age (year) | ≤60 | 25 | 3 | 28 | 2.408 | 0.121 |
| | >60 | 35 | 12 | 47 | | |
| Sex | male | 40 | 11 | 51 | 0.025 | 0.875 |
| | Female | 20 | 5 | 25 | | |
| Grade | I/II | 16 | 3 | 19 | 0.422 | 0.516 |
| | III/IV | 44 | 13 | 57 | | |
| T stage | T1/T2 | 12 | 1 | 13 | 1.701 | 0.192 |
| | T3/T4 | 41 | 13 | 54 | | |
| N stage | N0/N1 | 27 | 3 | 30 | 3.293 | 0.070 |
| | N2/N3 | 32 | 12 | 44 | | |
| M stage | M0 | 53 | 15 | 68 | 0.394 | 0.530 |
| | M1 | 7 | 1 | 8 | | |
| TNM stage | I-II | 21 | 3 | 24 | 1.595 | 0.207 |
| | III-IV | 32 | 11 | 43 | | |
| Tumor size | ≤5cm | 38 | 7 | 45 | 2.238 | 0.135 |
| | >5cm | 21 | 9 | 30 | | |
| Pathology Type | eminence-type | 9 | 1 | 10 | 5.676 | 0.059 |
| | ulcer-type | 25 | 3 | 28 | | |
| | Infiltration-type | 24 | 12 | 36 | | |

**Table S3. Differential expression of PTBP1 in cancer and adjacent tissues.**

| | n | PTBP1 expression | | Chi-square Value | p-value |
|---|---|---|---|---|---|
| | | **High** | **Low** | | |
| cancer | 76 | 16 | 60 | 32.089 | **<0.001** |
| Adjacent tissues | 82 | 1 | 81 | | |

* Statistically significant ($p < 0.05$)
