# Supplementary figures and images for: PTBP1 drives c-Myc-dependent gastric cancer progression and stemness
Source: Br J Cancer. 2023 Jan 12;128(6):1005–18. doi: 10.1038/s41416-022-02118-5 (PMC10006230; doi:10.1038/s41416-022-02118-5)

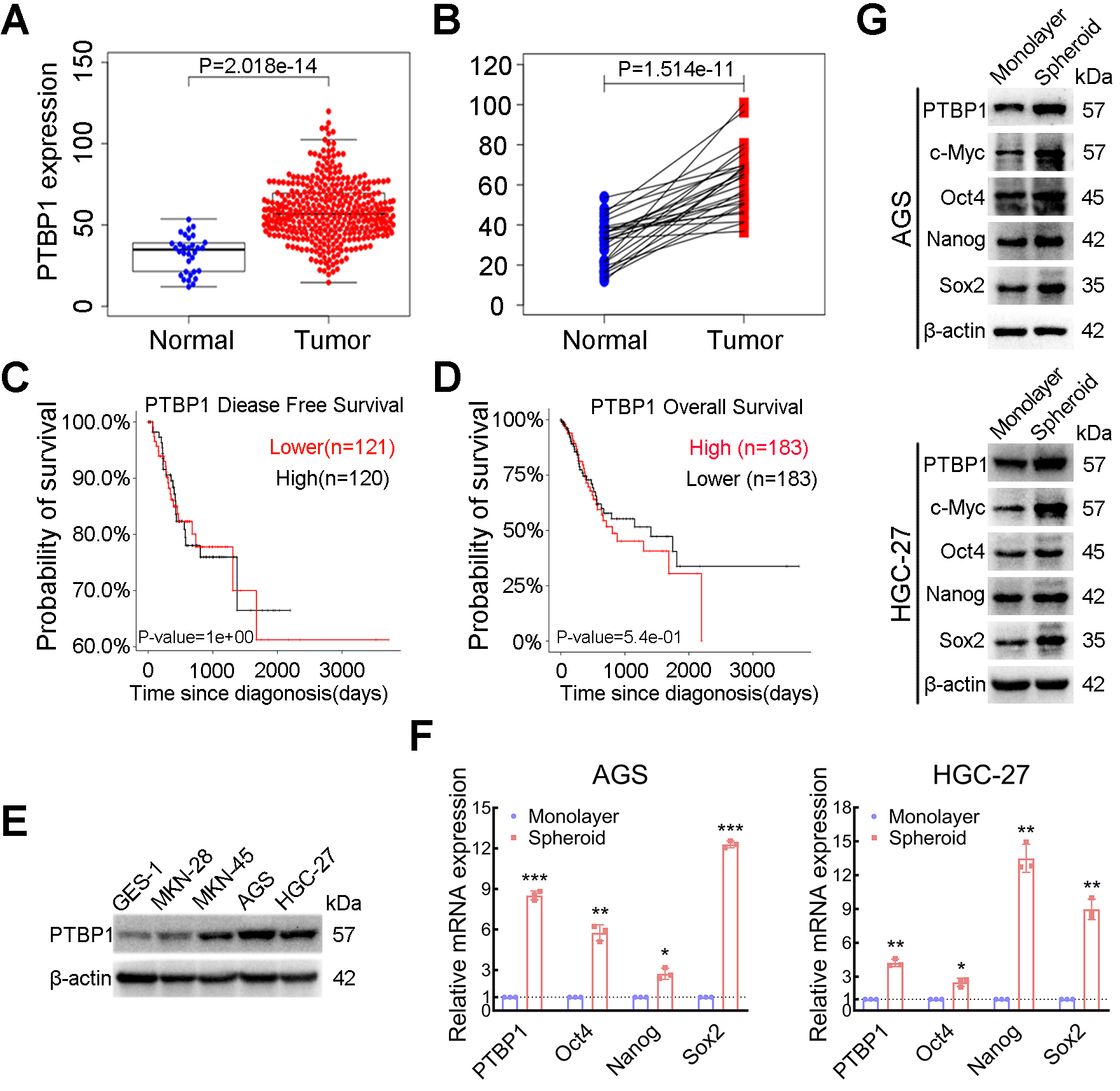

Supplement: Supplementary file 3 — Figure S1 [file 41416_2022_2118_MOESM3_ESM.tif]

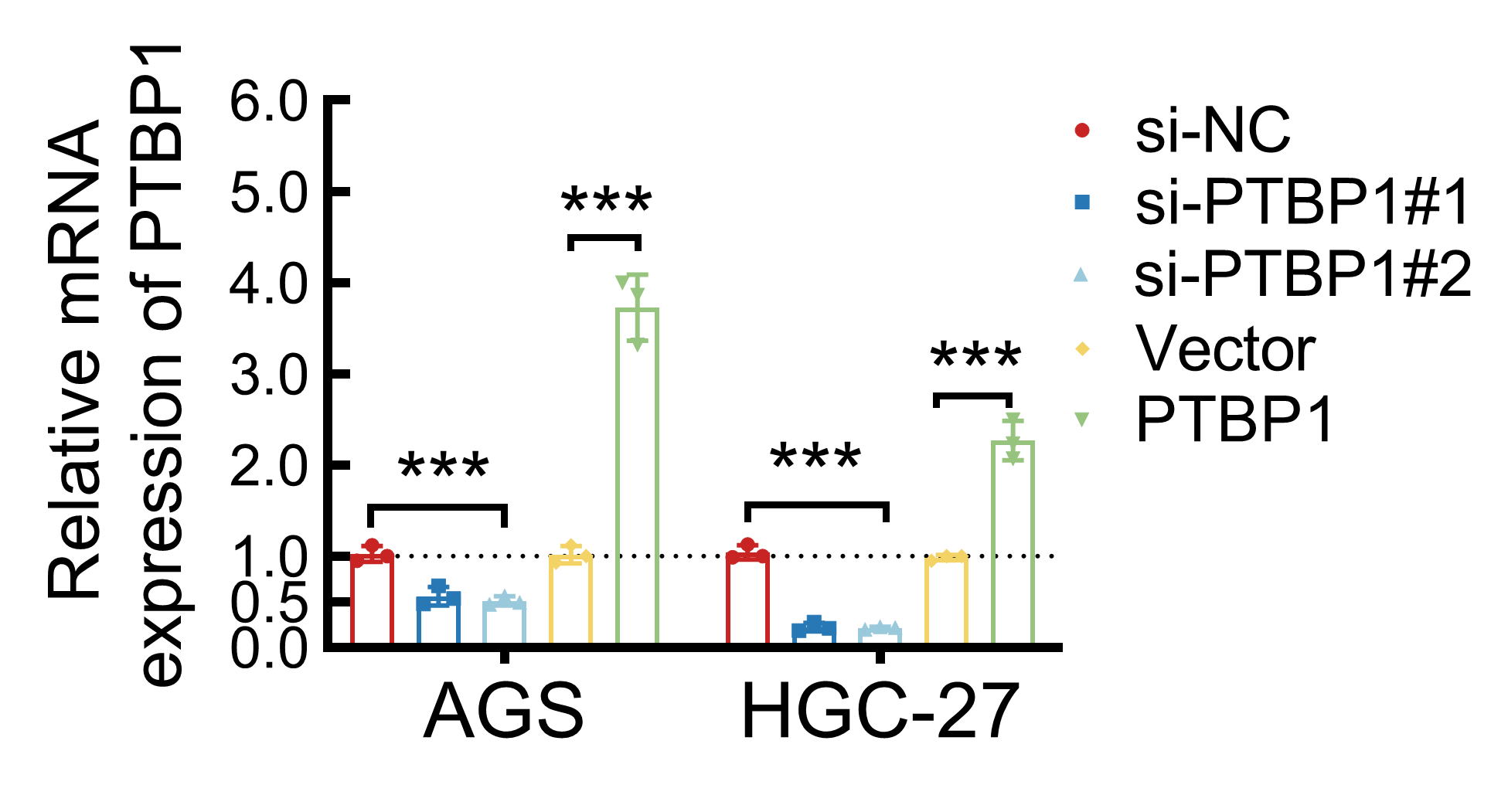

Supplement: Supplementary file 4 — Figure S2 [file 41416_2022_2118_MOESM4_ESM.tif]

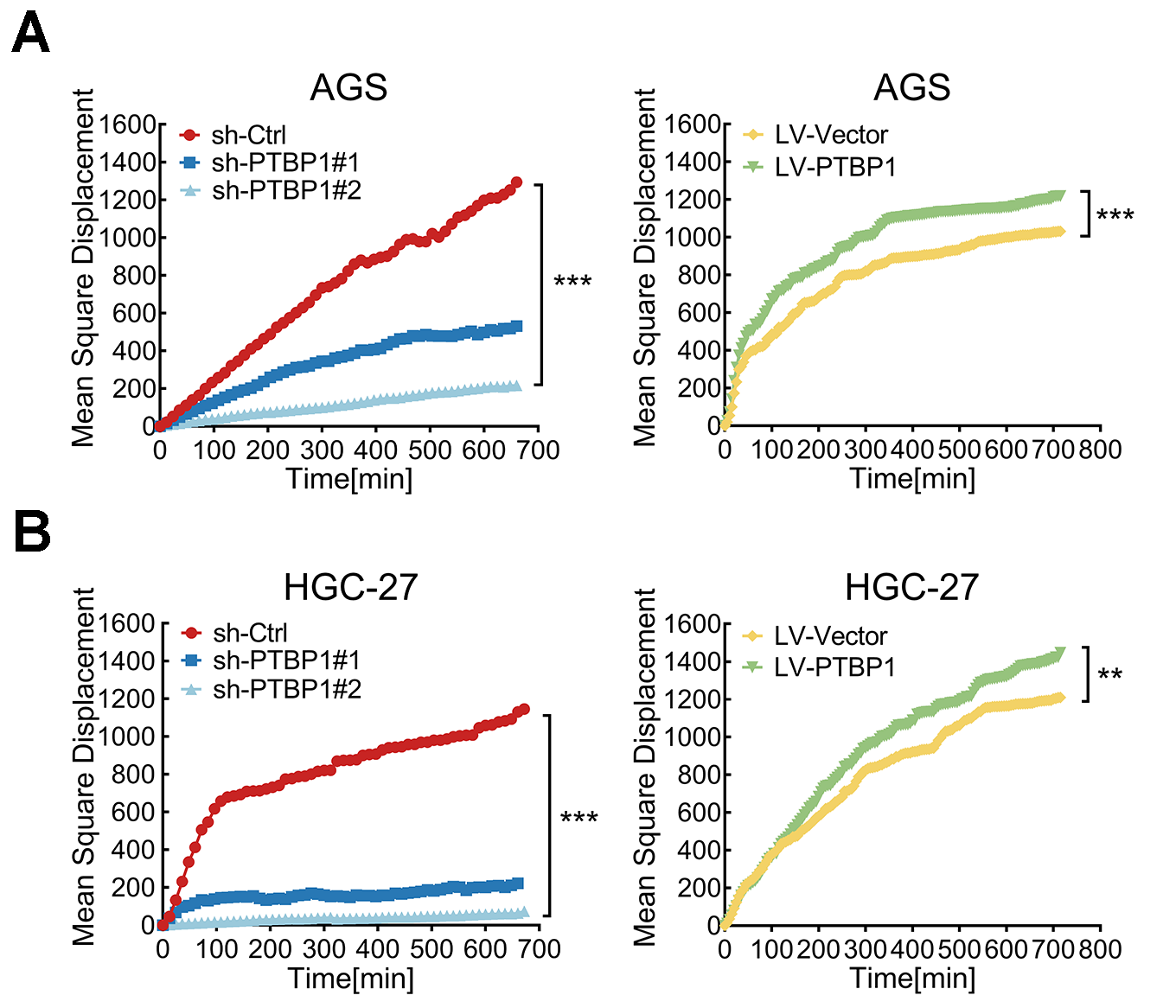

Supplement: Supplementary file 5 — Figure S3 [file 41416_2022_2118_MOESM5_ESM.tif]

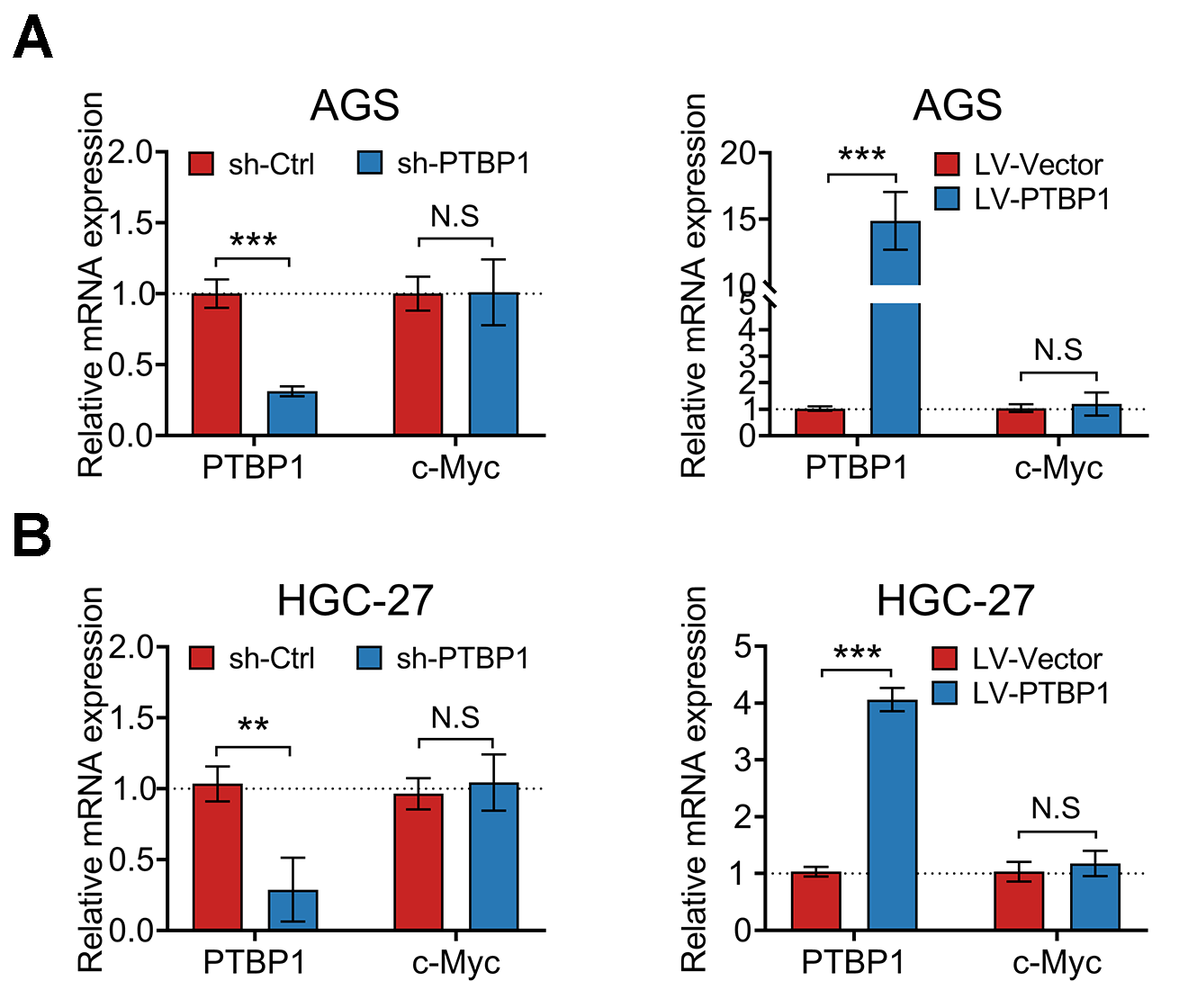

Supplement: Supplementary file 6 — Figure S4 [file 41416_2022_2118_MOESM6_ESM.tif]

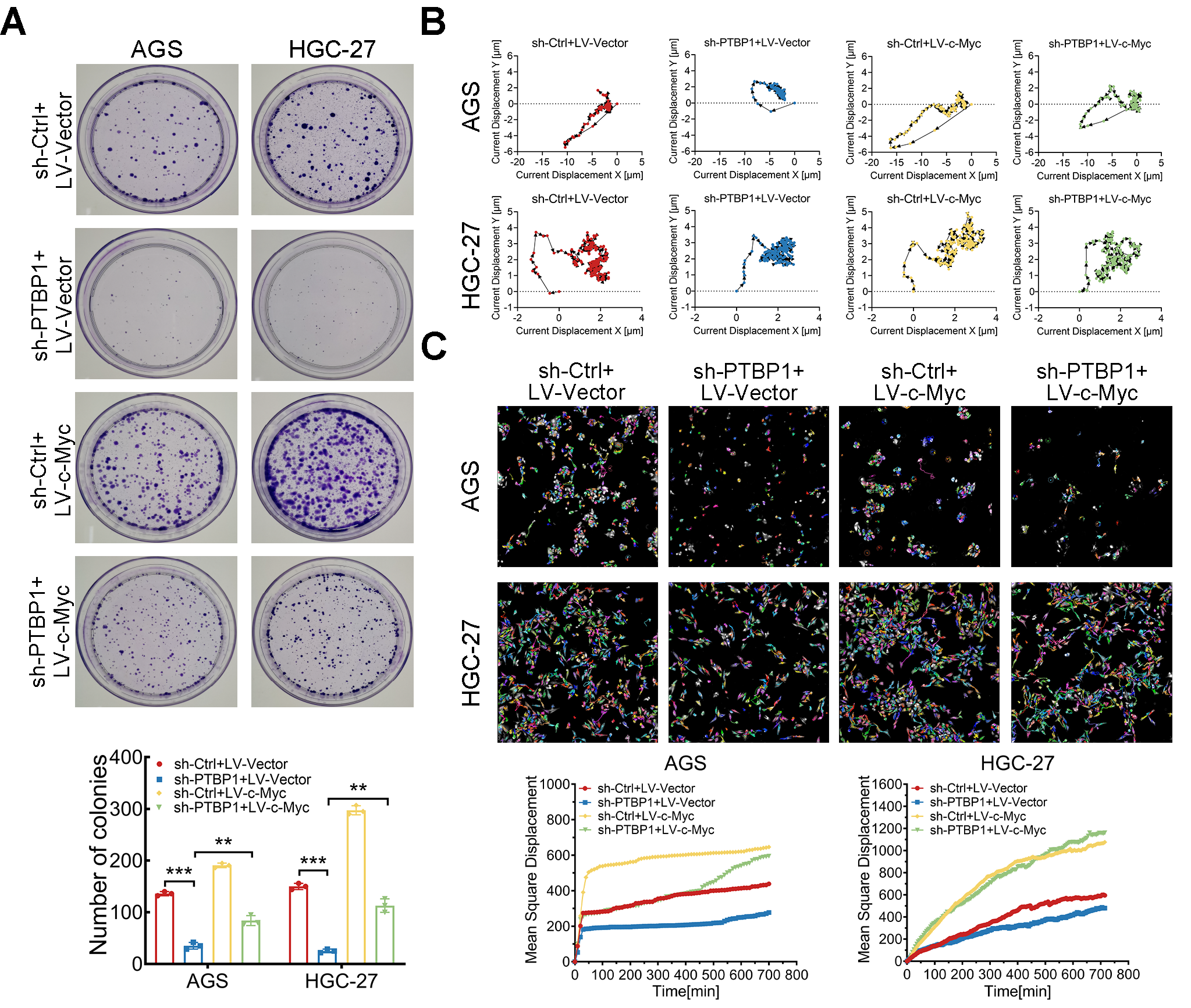

Supplement: Supplementary file 7 — Figure S5 [file 41416_2022_2118_MOESM7_ESM.tif]
